# How to design decision-support tools for primary healthcare using a human-centred design approach: the processes and experience of PHISICC in three Sub-Saharan countries

**DOI:** 10.1136/bmjgh-2025-019180

**Published:** 2026-01-14

**Authors:** Damaris Rodriguez Franco, Co-creation groups, Datte Atta K Sébastien, Christian Auer, Meike-Kathrin Zuske, Angela Oyo-Ita, Artur Muloliwa, Richard B Yapi, Salimata Berté, Mamadou Samba, Abdullahi Bulama Garba, Anthonia Ngozi Njepuome, Nnette Ekpenyong, Graça Matsinhe, David Brown, L Kendall Krause, Jahit Sacarlal, Xavier Bosch-Capblanch

**Affiliations:** 1 Sonder Collective, Madrid, Spain; 2 Swiss Tropical and Public Health Institute, Allschwil, Switzerland; 3 University of Basel, Basel, Switzerland; 4 Department of Community Medicine, University of Calabar Teaching Hospital, Calabar, Nigeria; 5 Centro de Estudos Interdisciplinares Lúrio, Universidade Lúrio, Nampula, Mozambique; 6 Centre Suisse de Recherches Scientifiques en Côte d’Ivoire, Abidjan, Lagunes, Côte d’Ivoire; 7 Centre de Recherche en Écologie, Université Nangui Abrogoua, Abidjan, Abidjan Autonomous District, Côte d’Ivoire; 8 Ministère de la Santé, de l’Hygiene Publique et de la Couverture Maladie Universelle, Abidjan, Côte d’Ivoire; 9 Université Felix Houphouët-Boigny, Abidjan, Abidjan Autonomous District, Côte d’Ivoire; 10 National Primary Health Care Development Agency (NPHCDA), Abuja, Nigeria; 11 Independent consultant, Abuja, Nigeria; 12 Population Services International (PSI), Luanda, Angola; 13 BCGI LLC / pivot-23.5°, Chapel Hill, North Carolina, USA; 14 Gates Foundation, Seattle, Washington, USA; 15 Department of Microbiology, Faculty of Medicine, Universidade Eduardo Mondlane, Maputo, Maputo City, Mozambique

**Keywords:** Health services research, Health systems, Decision Making, Global Health, Interdisciplinary Research

## Abstract

**Introduction:**

Healthcare delivery should be based on evidence-informed decisions in the clinical, public health, managerial and policy domains. Data are gathered at the point of care via routine health information systems (RHIS). The Paper-based Health Information Systems in Comprehensive Care (PHISICC) project shifted the paradigm from data collection to decision-making, especially decision-making at the point of care by the frontline health workers. We used a human-centred design (HCD) approach to re-design a RHIS that is responsive to the needs of frontline health workers.

**Methods:**

The PHISICC research programme took place in Côte d’Ivoire, Mozambique and Nigeria and included the design and testing of a suite of paper-based RHIS tools. We report here the results of the HCD process. This was structured into three phases: (1) setup of co-creation group, (2) concept exploration and (3) detailed design phase.

**Results:**

The concept exploration included a brainstorming session and produced ‘quick paper mock-ups’, such as ideas to follow-up patients’ healthcare. The output of this ‘concept workshop’ was a design hypothesis of the health information system. A follow-up workshop identified the healthcare areas to prioritise. The first round of design developed a version of several tools. The second round consisted of user testing in the three countries. Several iterations were implemented, incorporating health workers’ feedback. Tools were pilot-tested and then produced and distributed for use in a cluster randomised controlled trial.

**Conclusion:**

The design phase of PHISICC combined HCD with clinical and public health domains. RHIS should be designed by qualified designers, content experts and users who focus on aiding decision-making of frontline health workers, applying a user-centred approach, from problem identification up to solution testing, multidisciplinarity, flexibility, teamwork and trust. We call for researchers, designers, healthcare providers, healthcare authorities and funding agencies to propose and pilot quality standards for the implementation and reporting of HCD in global health.

**WHAT IS ALREADY KNOWN ON THIS TOPIC**

- Routine health information systems (RHIS) are mainly used to collect health services data and to report them to higher levels of the health system.
- Health workers experience a heavy workload from data collection, hardly being able to use data that are conceived for reporting and not for decision-making at the point of care.
- Human-centred design (HCD) approaches can be used to bring new solutions to old problems.

**WHAT THIS STUDY ADDS**

- A thoroughly conceived and carefully implemented HCD process can produce new RHIS tools that are a better fit for purpose and accepted by its users.
- HCD can operate a paradigm shift, where RHIS are understood as key components of quality healthcare as opposed to mere instruments to collect and report data.

**HOW THIS STUDY MIGHT AFFECT RESEARCH, PRACTICE OR POLICY**

- Implementing agencies working on RHIS, whether digital or paper-based, should work in close collaboration with qualified and well-experienced designers, from problem formulation up to solution design and testing.
- Policy-makers should demand that RHIS primarily serve to support clinical and public health decision-making and also to report data to higher levels of the system, without diverting health workers’ efforts from healthcare tasks.
- Research standards to carry out research on RHIS using HCD are urgently needed, to produce robust results that can eventually feed into evidence syntheses for policy-making.

## Introduction

 Healthcare delivery should be based on evidence-informed decisions in the clinical, public health, managerial and policy domains.^1^ One of the components of the evidence base for clinical care and public health consists of data gathered at the point of care: routine health information systems (RHIS) encompass the whole set of tools and processes used to collect and transmit information documenting the encounters between users of health services and healthcare providers, from the point of care, through the higher levels of the system, up to the national level.^2^ Hence, RHIS are inextricable components of healthcare processes and, as such, key contributors to quality of care,^3,5^ because diagnoses, treatments and referral decisions require understandable, accurate, complete and up-to-date information. In their role to support healthcare, RHIS can be conceived as healthcare tools, rather than merely support mechanisms for data collection and transmission.

Typically, healthcare workers record information about individual healthcare delivery activities, as they take place, in RHIS tools, for example, the name, contact details, clinical signs, diagnoses and treatments of a sick child or the vaccines given to a child and the date of administration taking into account his/her age and vaccination history. This information can be collected in the form of clinical notes or using facility registers, in paper, digital or hybrid supports. At the primary healthcare (PHC) level in low- and middle-income countries (LMICs), it is more common to find paper-based registers (ledgers) than individual or family records, because the former are easier to handle and require less physical storage space than the latter. Paper registers are typically produced at central level in tabular forms, where rows are used to list patients and columns to collect certain information about them.

Attempts to adapt and eventually improve the performance of RHIS to date tend to consist of adding or disaggregating data items as new columns in registers or new data fields, although it may take a long time and funding for registers to be adapted, printed and distributed. For example, new fields are included to record data on new vaccines introduced to vaccination schedules (eg, COVID-19) or new tuberculosis (TB) regimens or to facilitate disaggregation by sex in summary statistics. This approach does not actively consider usability, generating numerous inefficiencies,^6^ in paper as well as in digital tools; furthermore, these forms traditionally ignore the potential of visual language in human-data interactions,^7^ which altogether may render these efforts sterile.

Another approach has been digitising components of the RHIS (eg, electronic immunisation records), in an attempt to comply with increasing demands of performance monitoring indicators from programmes and donors, while trying to maintain efficient recording and reporting processes.^8^ In LMICs, digitisation has been largely implemented at intermediate levels of the systems (eg, at district health management level with the widely used district health information system (DHIS)^9^) and much less commonly at facility levels, particularly in rural PHC facilities, due to limitations in the electrical supply, connectivity, digital literacy, infrastructures and equipment.^10^

Decades of research and investments in health systems seem to have failed to address some important issues related to the design, functionality and technicalities of RHIS,^11^ with the potential of compromising the very decision-making processes they are meant to support and, therefore, the outcomes of such decisions related to the health status of populations and health systems performance. Anecdotal but consistent evidence suggests that little progress has been made in the design and usability of RHIS tools, while renewed efforts are dedicated to building the case for RHIS investments.^12^ Digital tools do not seem to fulfil the expectations generated,^13^ and health workers remain overburdened with data collection and reporting tasks.^14 15^

Cognisant of these issues and of the fact that digital solutions are not the panacea to address them, the Bill & Melinda Gates Foundation issued a call for proposals to carry out research on paper-based health information systems. The Paper-based Health Information Systems in Comprehensive Care (PHISICC) research programme^16^ (2015 to 2021) was implemented in Côte d’Ivoire, Mozambique and Nigeria, in close collaboration with research institutions and ministries of health. The objective of PHISICC was to produce a paper-based system using a human-centred design (HCD) approach (the intervention). PHISICC operated exclusively at primary healthcare level and used a tally system to link with the DHIS2 reporting (DHIS2: District Health Information Software 2). While the three countries have similar RHIS reporting structures and periodicity, from the periphery of the system up to national level, each has some specificities in terms of the amount and types of information collected and reported. For example, the number of items inventoried from the monthly reports is more than 1500 in Côte d’Ivoire, almost 500 in Mozambique and a bit more than 600 in Nigeria, reflecting a diversity in the number of data items requested or their level of aggregation.

The critical breakthrough in the PHISICC approach was to focus on decision-making of frontline health workers and realising that any attempt to improve RHIS should focus on their decisions in their daily, routine practice. These were primarily clinical and public health decisions, such as ‘treat on the spot or refer’, ‘vaccinate now or delay vaccinations’ or ‘how to best promote the use of mosquito nets in my community’.

Strategic and policy decision-making requires good quality data, as well as appropriate cognitive information processing^17^ and a good understanding of the mechanisms underlying policy-making,^18^ among other aspects. However, traditional RHIS improvement approaches consider RHIS as monolithic entities typically serving management functions, as well as the data needs of programmes, the international community and global initiatives.^19^ How these data needs are made compatible with the overwhelming clinical and public health workload of frontline health workers, particularly in remote, rural areas, has been largely neglected. We hypothesised that RHIS require different subcomponents serving different decision-making domains.^1^

Given the paucity of evidence on interventions to improve paper-based tools and the overall perception about the limitations of approaches focusing on data rather than on decisions, we engaged in an HCD process to create a new suite of paper-based tools to support health workers’ decision-making in remote, rural PHC settings.

The introduction of HCD in domains where it has not been traditionally used has brought new expectations to build appropriate and patient- and health worker-centred solutions to healthcare and health systems problems.^20^ ‘Human-centred design (HCD) is a problem-solving process that begins with understanding the human factors and context surrounding a challenge. It requires working directly with users—the people who use the service or deliver the solution—to develop new ideas that are viable and appropriate in their context. Designing for people and their everyday actions helps uncover and solve the right problems using local capacities and minimal resources’.^21^ In other words, HCD ‘is an approach that aligns innovation development with the needs of the people and the settings where those innovations will be used’.^22^

The human-centredness comes from the fact that HCD looks at problems from the perspective of those experiencing them in their real lives and work situations, designing solutions from the perspective of those who are going to implement them. A growing body of evidence suggests that HCD could be an opportunity to improve health outcomes as it can ‘help the health community shift from prescribing solutions according to a perception of people’s needs, to identifying solutions that actually meet their needs’.^23^

The objective of this article is to document how HCD, combined with public health and clinical medicine, produced a paradigm shift in the understanding, ideation and design of RHIS for remote, rural PHC facilities that truly serve as a decision-making instrument to improve quality of care and not only as data collection tools.

## Methods

The PHISICC research programme took place between 2015 and 2021 in three Sub-Saharan African countries: Côte d’Ivoire, Mozambique and Nigeria. Following the 11 principles of transboundary research partnerships,^24^ PHISICC was conceived as a joint effort of teams with complementary skills and commitments in ministries of health, health research institutions and in the design domain. The overall coordination across the three countries took place from the Swiss Tropical and Public Health Institute in Switzerland. The team in Switzerland was composed of public health experts, specialists in experimental study design and a senior statistician. In each country, a memorandum of understanding was signed with the corresponding Ministry of Health, in support of the development, implementation and testing of an innovative paper-based information system in primary healthcare. This also provided the necessary administrative and ethical clearances and facilitated the involvement of ministry counterparts in strategic decision-making during the life of the project. A contract was signed with a research institution in each country to carry out the research. The generic protocol contained a common rationale, objectives, interventions and outcomes; and it was adapted to the three countries in terms of language, timelines and information items, among other issues. Researchers in the countries were a mix of public health experts, social scientists and an anthropologist in Côte d’Ivoire. The PHISICC research programme was supported by a Technical Advisory Group (TAG) that provided support and monitored progress. The TAG consisted of a mix of professional profiles, including experts in evidence synthesis, multilateral organisations and global health initiatives and the PHISICC funding agency. This quadripartite arrangement (Swiss Tropical and Public Health Institute (Swiss TPH), ministries of health, research institutions and TAG), together with agile and periodical exchanges between parties and shared decision-making, provided the platform that guided the project from its start until the end. For example, while the contents of PHISICC forms were guided by health experts from each country, design issues were mainly responsive to the iterations with health workers, as reported in this article.

The research programme involved six work streams (WS): 1, inception phase to set up the project management and technical protocols; 2, research syntheses^25^ to learn what others did before us; 3, characterisation of existing RHIS in the countries, to understand what was already in place; 4, ideation, design and production of the paper-based intervention; 5, testing the intervention in three cluster randomised controlled trials;^26^ and 6, advocacy and dissemination. Further details on the aims, setup and phases of the PHISICC research programme are reported elsewhere.^16^ We report here on WS4, the intervention design process.

The design partner was selected following an open competitive process. Criteria taken into account included: proven track record of using HCD approaches, experience in working in Sub-Saharan African settings, familiarity with multidisciplinary approaches, including scientific methods, willingness and capacity to travel to the countries, flexibility and adaptability to changes in management, practices and context.

**Figure 1:**
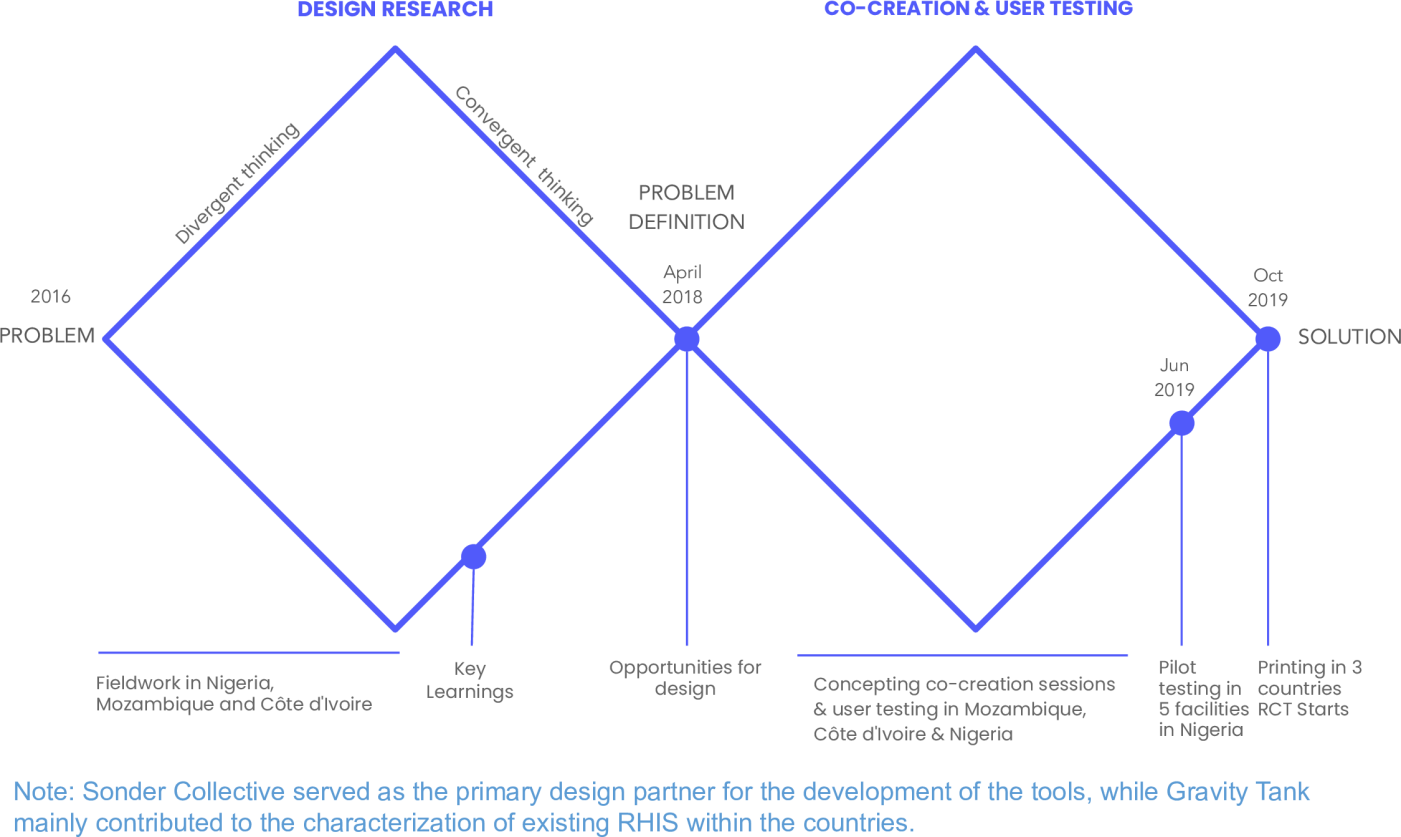
PHISICC design process. PHISICC, Paper-based Health Information Systems in Comprehensive Care; RHIS, routine health information systems.

The overall design process in WS4 is summarised in figure 1 and in table 1. The design process was structured with the aim to prioritise outcomes (value) over outputs (efficiencies). The process was flexible to meet the design team’s needs for field-testing and co-creation with teams formed in all three countries, if required. The design process included iterations (four in Côte d'Ivoire, four in Mozambique and six in Nigeria) involving co-creation groups, research teams and frontline health workers, facilitated by the design team to refine the final visual design. The iterations occurred from May 2017 to June 2019, with slight country-specific adaptations. Online supplemental 1 illustrates some of the processes of the field work.

**Table 1:** Summary of the design phases

	1. Co-creation group set up	2. Concept exploration phase	3. Detailed design phase
Duration	3 months	4 months	12 months
Activities	Select design partner for co-creation phase Setup co-creation group in the three countries Launch co-creation work stream workshop in Côte d’Ivoire	Concept workshop week Concept testing week in Mozambique First design round User testing round conducted by the health workers	Second design round: high fidelity prototypes* of all healthcare areas User testing field trips in Côte d’Ivoire and Nigeria Nigeria two weeks pilot testing Local production of all the tools for the trials in Côte d’Ivoire, Mozambique and Nigeria
Outputs	Design company engaged Co-creation groups trained	Key research findings Opportunities for design Low-fidelity prototypes: sick child, antenatal care, vaccination	Final designs of all healthcare areas registries and tallies (antenatal care, delivery, post-natal care, vaccination, sick child, outpatient department, HIV, tuberculosis, referral)

* High-fidelity prototypes are advanced mock-ups that closely resemble the final design in both appearance and functionality.

The intervention design stream was led by the design partner who deployed its experience in service and product design using HCD approaches in Global Health. The HCD approach required setting up co-creation groups that would take responsibility for the ideation and design of the intervention. Co-creation refers to the collaborative process of creative problem solving between diverse stakeholders at all project stages. It emphasises the importance of engaging diverse stakeholders at all parts of the process, from problem definition through the project’s conclusion.^27^

The co-creation groups focused on frontline health workers from areas similar to, but outside, the study areas to prevent intervention contamination during the trial phase. Additional members from ministries of health and research institutions were included to provide necessary expertise and ensure ownership of the intervention. We took active measures to capture voices that would normally be relegated, such as breakup groups, individual discussions and on-site visits.

The criteria for the selection of co-creation group members included: first-hand experience in providing healthcare in remote, rural areas; good knowledge of the RHIS used in remote rural areas; capacity to dedicate time over months to try the tools and provide feedback to the design team; capacity to travel for workshops; and willingness to participate and explicit authorisation from supervisors. The team encouraged the participation of health workers and researchers from remote areas. We established a co-creation group in each country, totalling 10 members, which worked together for 16 months, though mostly in their respective countries.

Governmental partners participated in the selection of the co-creation groups and the final selection was agreed with the research team. The initial training was carried out in Côte d’Ivoire. During the training, designers built capability in the co-creation methodologies. Ten people from the three countries were trained in co-creation HCD approaches and learnt to conduct user testing by the senior designer in the research team. Training consisted, for example, of a mix of simulated form completion exercises and usability testing and iterative feedback with prototypes.

The piloting was carried out in five health facilities in Nigeria. These were purposively selected from health facilities outside the study area, with some variability in terms of size and volume of activity, and readily accessible by the research and design teams.

### Patient and public involvement

In all three co-creation groups, at least one of the members was a frontline healthcare worker. The development of the prototypes was led by designers, who translated the co-creation group’s contributions into the high-fidelity prototypes that were subsequently tested with users. Prototypes of the emerging health information tools were repeatedly field-tested among frontline healthcare workers, and their feedback was carefully considered and incorporated into the next version of the emerging tools. We did not specifically ask patients for feedback; however, during piloting, the healthcare workers used the pilot PHISICC tools while interacting with patients.

The overarching aim of WS6 was to ensure that the research was relevant and responsive to the needs of partner countries and the recipients of the PHISICC tools. To this end, from the very conception of the project, active engagement with the governmental health sector, research partners and other important stakeholders to facilitate their inputs was paramount. The profile of PHISICC was built through a series of communication and advocacy activities.

## Results

In this section, we report chronologically the progress and results of the co-creation processes alongside WS4.

### Concept workshop week

This phase began with a 5-day intensive workshop (‘Concept week’) involving the core team and two designers to synthesise critical challenge areas and develop opportunity areas for WS4. The team extracted the key findings generated in the WS3 characterisation of the RHIS and mapped them into a previously developed research synthesis framework.^25^

**Figure 2:**
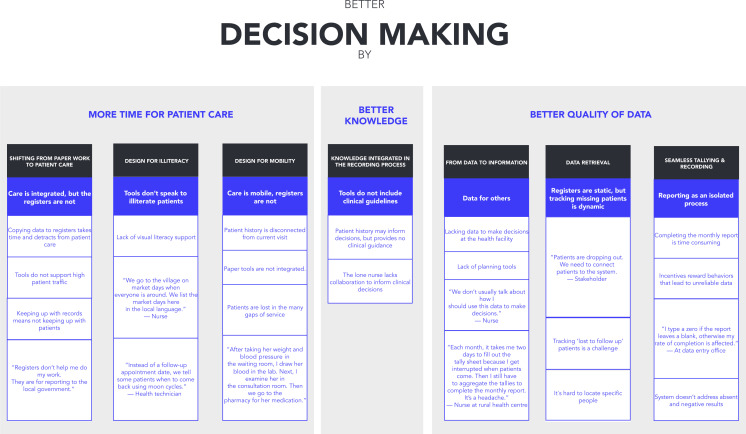
Key barriers and opportunity areas for a better health information system.

After the synthesis, the design and research team translated the key barriers identified into opportunity areas, based on the cross-country findings of WS3 and on existing research synthesis on RHIS. This step provided an understanding of the range of potential requirements of a RHIS, based on health workers’ experience and context of use (eg, health facilities with high volume and low volume of activities) (figure 2).

During the workshop, the design team facilitated an ideation session. Participants started to elaborate some of the ideas into ‘quick paper mock-ups’, such as ideas to follow-up users’ healthcare processes (eg, for successive consultations or vaccinations) and explorations for the vaccination calendar options. During the ideation phase, the design team presented initial concepts, while participants contributed additional ideas that were collaboratively refined and translated by the designers into prototypes for user testing. All prototypes were translated and tested in the corresponding language of each country.

**Figure 3:**
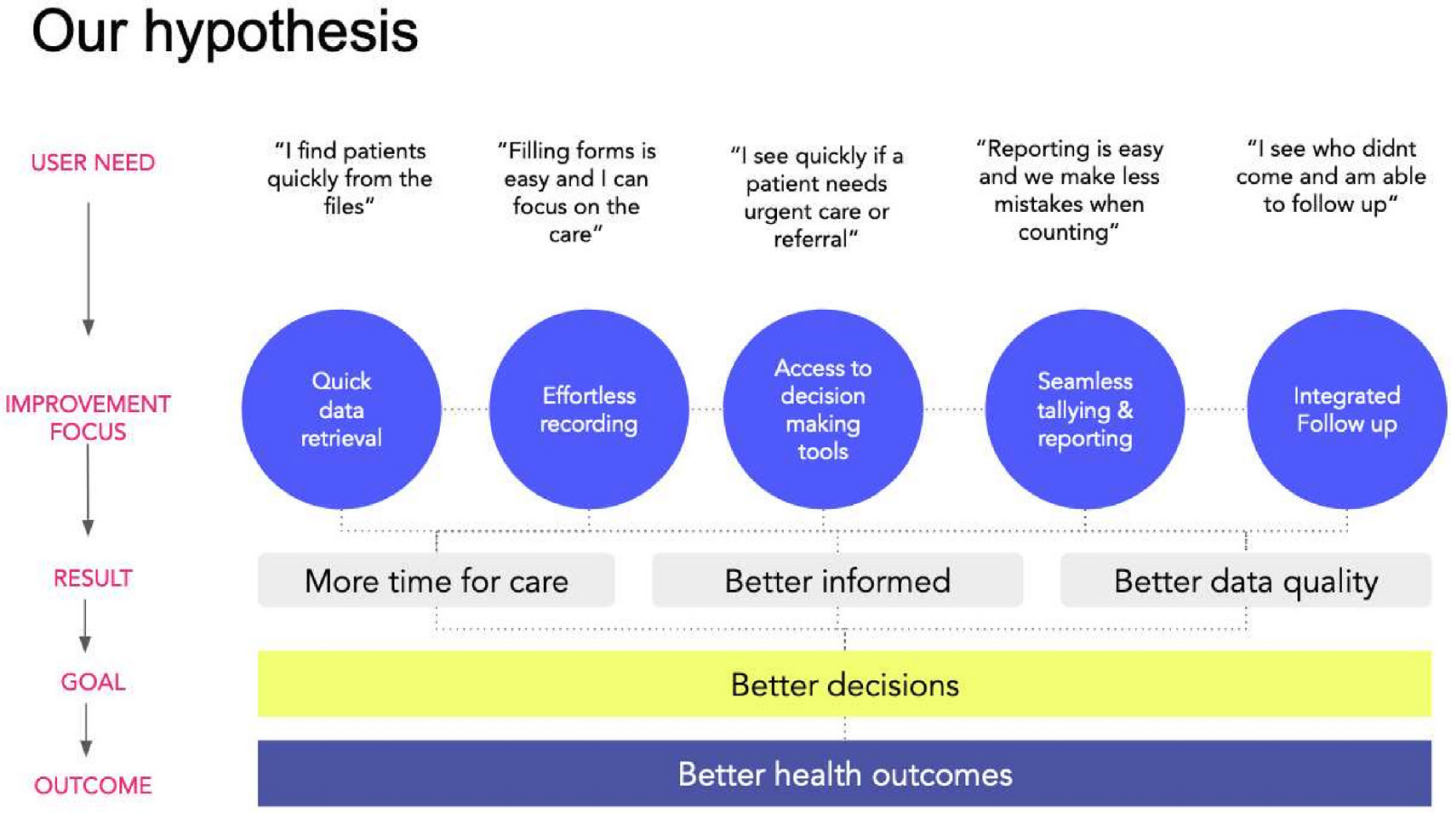
Design hypothesis related to the health information system.

The output of this ‘concept workshop’ week was a design hypothesis of the health information system (figure 3), grounded in all the work developed throughout WS2^25^ and WS3 and in the PHISICC framework. The hypothesis is based on the consolidated key users’ needs and shaped into five requirements for RHIS innovation areas: (i) quick data retrieval, (ii) effortless recording, (iii) access to clinical guidelines and aides, (iv) seamless tallying and reporting and (v) integrated follow-up.

Quick data retrieval was related to finding a mechanism that would allow health workers to retrieve users’ past information, such as vaccination history status or previous antenatal care consultations (eg, writing in the home-based record the book and page where the corresponding complete antenatal or vaccination data for that individual was recorded). Effortless recording pointed at reducing the effort to fill in the relevant data items during clinical or other healthcare encounters. Access to decision-making tools referred to the capacity to access information that is important for healthcare delivery, such as treatment guidelines, anthropometric scales and alike; indeed, the questions in the recording tool that guided the health provider towards the correct diagnosis were based on clinical guidelines. Seamless tallying and reporting was an innovation consisting of providing tally sheets with the same monthly report indicators for each healthcare area, to be used at the time of each provider-user encounter. The left part of the sheet contains the indicators and for each indicator small ovals to tally, and the right side contains cells to write the counting and is separated from the left side with a perforated line. Tallying is often done for vaccinations but not for other healthcare areas. If tallying is completed when patients are seen, then, at the end of the month, when the monthly reports to send to higher levels of the systems are due, there is no need to spend all that time browsing again through all registers; rather, health workers just complete counts in the tallies and send out to the higher level the right part of the tally sheet. This change in the reporting format was made in such a way that all the reporting requirements for DHIS2 were still included in these tally/reporting sheets. Integrated follow-up referred to mechanisms to easily identify patients or users requiring follow-up consultations. All these were specific issues that heavily worried health workers and which had a high potential for innovation.

### Co-creation week with the three countries

After the concept week, the design team generated a first version of the vaccination and sick child registries. These two healthcare areas were prioritised because they (i) are well standardised across countries; (ii) are supported by guidelines; (iii) are good examples of public health (vaccination) and clinical (sick child) interventions; (iv) offer a good variety of situations (sick child); and (v) have a strong component of follow-up (vaccination).

The PHISICC research team, in-country co-creation groups and stakeholders convened in Maputo, Mozambique, for an intense week of co-creation and the first round of user testing. More than 20 co-creators and project stakeholders came together in the course of a 5-day process. The week’s goal was to validate the findings of the concept week, create alignment, explore ideas together, iterate the prototypes, conduct the first round of user testing together in the field and make decisions for the design. This was also an excellent opportunity for cross-learning among the three countries.

In this process, several critical choices emerged, including (i) the generalised use of tallies across all healthcare areas and (ii) use of registry books format over individual patient records. The tool design’s critical decisions were to co-decide the type of registry data retrieval (ie, semi-bound to enable retrospective filing; one register per year/quarter/month, one or several clients per register page and separate registers for each healthcare area).

Through the co-creation sessions and the field visits to health facilities, the co-creation groups decided to keep the existing registry book concept, emphasising the innovation efforts in the decision-making experience, while minimising the impact of a complete shift by introducing a single page or semi-bounded register concept. The focus on decision-making in clinical care (particularly for the sick child) was one of the concepts discussed at length, and the co-creation group aligned on the need to push the boundaries of the first set of prototypes, adding more visual tools to facilitate or guide the health workers’ decision-making.

### First round of design

The design team took all the learnings from the co-creation week and developed a high-fidelity version of tools for other healthcare areas as well: maternal health registries (ie, antenatal care, delivery and post-partum), sick child, vaccination registries and tallies. The research and the design teams identified the key elements in clinical guidelines that would meet frontline health workers’ decision-making needs and transferred those items into a meta-data file. The process was very iterative, and the tools were continuously evolving with the feedback of the co-creation groups. The first round of the design was followed by a second round of user-testing in the three PHISICC countries. The testing aimed at learning, developing and iterating the current tools developed in the areas of vaccination, sick child and maternal health. Testing was conducted by each country’s co-creation group to ensure contextual adaptation and value for frontline workers. The design and research teams joined the co-creation groups in the fieldwork activities in Côte d’Ivoire. The field visits were conducted in 12 health facilities (ie, four in Côte d’Ivoire, three in Mozambique and five in Nigeria). The co-creation group members went to the field and tested the latest developments with the frontline health workers and asked them to populate them with real information. After completion, health workers identified challenging data fields and provided suggestions for improving subsequent versions.

The emerging learnings were used to improve the clinical terminology to be adapted to the context, adjustments to the slightly different vaccination schedules (eg, Inactivated Poliovirus Vaccine (IPV) in Mozambique at 14 weeks) and data that were missing in some forms. The team also started to evaluate the usability of the tools (eg, some fields needed more space, typeface sizes) and the perceived value from healthcare workers (eg, ‘it gives direction and eases stress’ was mentioned by a frontline health worker in Nigeria).

Based on collaborative insights from co-creation groups, the design team defined the overarching concept for executing tools across remaining registries and tallies in other healthcare areas.

### Second round of design

The design team worked with the research team to develop the remaining registries and tallies for outpatients (OPD), HIV and TB. The process involved discussing with clinical and public health experts to embed the clinical protocols and current country practices into the registries. Simultaneously, the research team worked with the statistics offices of ministries of health at district and central levels and with the in-country research partners to validate the healthcare indicators in the tallying/reporting subsystem that would feed the existing systems from district level upwards (eg, DHIS2).

The second round of design was supported by a co-creation week in Nigeria, where health workers were invited to a day-long workshop to test the tools and provide a deeper level of contributions. A group of two sets of four to six frontline health workers was invited to simulate different scenarios of use of the tools, performed the simulation activities and reflected on the experience. The design team and researchers were able to see in action how the frontline workers were learning the tools, which data were easier or more difficult to use, the time to enter the data and typos identified by the health workers.

**Figure 4:**
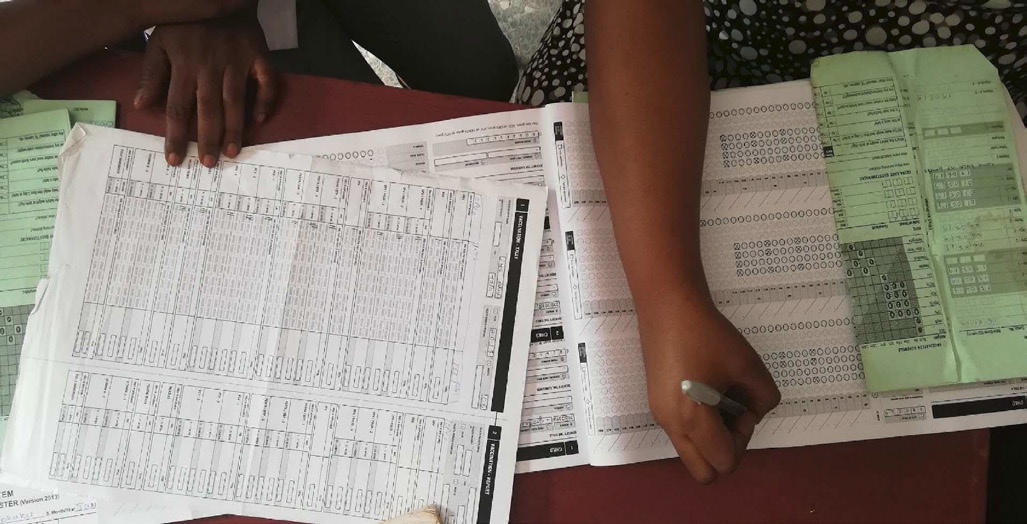
Design team elaborating on vaccination tools during vaccination day.

The emerging learnings from this week in Nigeria revealed that the new forms highlighted more their clinical weaknesses: the terminology used in the forms, where healthcare workers exhibited a lack of comprehension, frequently revealed deficiencies in their familiarity with clinical guidelines. There was also a rethought on how to create better linkages between the HIV registry and the different healthcare areas. The design and research teams worked very closely to develop the tools’ next iteration (figure 4). The new iteration was piloted in healthcare facilities in Nigeria for several weeks. The fact that the contents of the forms were mainly clinical facilitated the standardisation across countries. All versions of the forms were tested across the three languages during development, allowing us to adapt content in real time. We prioritised using simple, accessible language that would be easily understood by junior staff or participants with lower literacy levels.

### Pilot testing in Nigeria

The pilot’s goal was to test the tools in the facilities with clients to ensure the tools (ie, namely the new registers) were ready for the testing in real life situations. The design team visited the facilities three weeks after the PHISICC pilot tools had been introduced to learn from the frontline workers their experiences using the tools and to identify potential unintended consequences and the overall perceived value from the frontline workers.

During a week, the design and research team conducted observations and discussions across the selected health facilities during immunisation days and discussed with seven frontline workers their experiences, challenges with the new tools and things to improve in the next version.

The emerging learnings from the visit were that (1) health workers perceived the new system as easy to use compared with the previous system; (2) tools were perceived as a guide to the clinical encounter and provided mental structure to ask the right question in the right moments to patients and enter the data; and (3) data missing in the records were mainly due to health worker knowledge gaps (eg, the gestational age by calendar and signs of fetal distressing were often not filled in because of lack of familiarity of health workers with those measurements).

### Production and distribution

With the final recommendations from the frontline workers, the design team completed a final iteration of the tools. Online supplemental 2 contains some of these tools (immunisation and sick child), and online supplemental 3 shows several versions of the sick child form to provide an example of design changes over time. Nigeria was the first country to start the study to test the forms. Then, the final tools were translated from English to Portuguese and French, with their specific customisation based on countries’ needs and health indicators.

The design and research team produced 57 pages of the paper-based information system in three languages. The paper-based information system works across antenatal care, deliveries, postnatal care, OPD, sick child, vaccination, HIV, TB and referral.

As indicated above, there were multiple iterations with health workers but also with clinicians and managers, resulting in a substantial number of versions of the forms produced (eg, around 20 versions per form, and the sick child register with over 100 versions). The registers are identical in all three geographical areas. All healthcare areas have the same data items, the only difference being the language translation, except for the vaccination schedules that were country specific.

## Discussion

PHISICC is, to our knowledge, the first attempt to systematise the design of a RHIS that is primarily rooted in the decision space of its users (ie, frontline health workers) and that embraces the whole scope of primary healthcare activities; furthermore, it has brought together an innovative design solution with strong and rigorous evaluation processes, combining HCD with the clinical and public health domains within an experimental study design.^26^

We have documented an HCD process that successfully engaged a large number of experts, with very diverse profiles, including frontline health workers, into a co-creative process to develop a suite of paper-based information tools for primary healthcare in Côte d’Ivoire, Mozambique and Nigeria. In public health programmes that implemented HCD, the user (client, provider or community) has agency in shaping more contextually appropriate solutions.^28^ We approached contextual issues through three studies characterising the health information systems in the three countries, from a public health and design perspective, and through the preparatory work of this design phase (unpublished reports available through the corresponding author). We judge that this experience was a great success based on the fact that the newly designed tools were compliant with the requisites defined by health workers themselves, their routine use, the informal feedback from health workers and the preliminary findings from complementary studies, using both quantitative and qualitative approaches.^29^

Given the limited evidence on the effects of the application of HCD in healthcare, we want to explore potential drivers of success and also highlight some risks. Very importantly, HCD produced a change of paradigm on how we conceived RHIS. In the beginning, the thinking of RHIS in the PHISICC research programme was focused on the availability and quality of data to be collected in health facilities and transmitted to the higher levels of the system, until the national level (ie, recording and reporting).^19^ But different types of decisions require different types of data.^30^ The HCD approach helped us to acknowledge that the keystone of frontline health workers’ mandate is to make sound clinical and public health decisions and not to collect and transmit data. Therefore, the focus was on developing a RHIS that supports health workers’ healthcare decisions. And because a RHIS that supports clinical decisions may not be perfectly suited to support other managerial decisions at health facility or higher levels, we had to find the solution of adapting the monthly reporting component of the RHIS, bridging clinically focused registers with monthly reporting with the seamless tallying process (it is important to note that new tools replaced the usual tools, to avoid increasing the workload of health workers).

HCD also allowed us to remain in a systemic perspective.^29^ While similar efforts to test RHIS interventions tend to be circumscribed to individual healthcare areas or programmes (eg, neonatal care, diabetes),^31^ we decided to embrace the whole set of healthcare delivered by frontline health workers in PHC settings, among other reasons because they are the same health workers who deal with all conditions and to avoid fragmentation, an issue that jeopardises the use of data for decision-making.^19^ This decision brought enormous complexities (eg, the fact that not all conditions have clear guidelines) but was a must to keep the newly designed tools relevant to its users.

The systemic perspective also allowed a deeper understanding of the technical requirements and constraints of the design solution. In conception, we say that constraints and limits are the sources of inspiration. From the beginning, it was clear that the intervention (the solution) had to be of minimal cost, so the teams from the beginning designed a solution that would be black and white and use standard paper sizes. The solution should be printed anywhere. These limitations early in the design process prompted the team’s creativity and forced the design solution to be very innovative on small details. The innovative design was a result of working with strong constraints and keeping the innovation grounded in the context.

The HCD process took a long time to complete, in line with the HCD experiences of others,^32^ which is a challenge for the reproducibility of this approach. The experienced challenges can be attributed to the relative novelty of the approach in global health,^33^ the scope and complexity of the PHISICC intervention and the comprehensiveness with which HCD was implemented. We implemented the HCD in a comprehensive manner: from the initial development of the research proposal up to the dissemination of findings and in each step of the problem identification and solution design. Ideation, innovation and problem solving in general require time to think, exchange, read, discuss and engage. For example, while every actor acknowledged (and welcomed) the need to simplify reporting, health workers themselves seemed reluctant to simplify the forms on the grounds of the directives currently in place; however, this had to be compatible with the usability of the tools, a design principle that could not be ignored. Handling these types of tensions required an extremely professional and committed team of designers and researchers to handle the power dynamics, as well. Furthermore, interactions across countries, with each one sharing its own experience, opened a way to resolve complex challenges in RHIS that we would not have been able to resolve in a single location. We leveraged best practices and learnings consistently across the three geographies, creating a systematic design solution.

HCD also brought some risks and challenges. HCD brought a point of uncertainty to the project because health workers’ contributions could not be predicted and we could not plan for a specific intervention from the outset of the research. Actually, this is precisely why the HCD process is set up: to discover what is truly important for users. Therefore, donors, researchers, designers and managers had to exert a great deal of flexibility. We are equally aware of the potential risks and pitfalls of HCD when not properly applied or when applied in certain domains.^34^

Interdisciplinarity played an important role throughout the process. The timings, methods, conceptual frameworks and implementation of HCD approaches collided with the rather rigid methods of public health and health systems research. There was a process by which the public health experts in the team progressively embraced the HCD approach. This was possible because of (i) optimal human relationships based on trust; (ii) sharing a common objective; (iii) giving time for the mental processes to take place; and (iv) the flexibility of the funder of the research to accommodate this process. As Mishra and Sandhu explain, the true value of design is fully realised when multidisciplinary teams work with communities to identify their needs, co-create and test ideas and facilitate decision-making.^35^ Despite the fact that the senior designer was a foreigner in the three countries, the relationship built over the months generated an environment of mutual trust.

Another challenge was the understanding of design partners of health workers’ needs and capacity. We worked with several design partners along the whole process that, despite the similarities in their remits, showed substantial differences in how HCD was to be implemented and how interactions in a multidisciplinary environment were set up. Some ambiguities and inconsistencies of the design terminology and methods can introduce confusion within an already complex process.

Finally, we also experienced the impossibility to modify certain issues that were, formally or informally, considered immovable in the existing health systems contexts. For example, while there is evidence of no sex difference in vaccination uptake, one of the countries was reluctant to remove the vaccination sex-disaggregated data, despite that it was not really used. It is difficult for us to systematise the success factors of such processes. The importance of having the health workers and their work at the very centre guided the whole process in every single step. The designers, researchers and co-creation groups were committed to elevating the frontline worker’s voice by carefully considering every piece of feedback with rigour, building long-term relationships with health workers and moving their role from participants to active partners. Their role was critical in the last iterations of the tools, keeping their input and voice to determine when the design was ready for use. We cannot rule out, though, whether another mix of members in the co-creation groups would have produced a less good or even a better result.

The co-creation process also allowed shared power in the research, decision-making and co-production of the tools, for example, whether to use registers or family folders, the reduction of redundancies in registers or data items, formatting issues such as sizes, colours and shapes. We expressed this as follows: ‘this tangible, people-centred focus on interventions also shifted the team’s own perspectives, from the more traditional system-wide, top-down analysis of health data, data collection processes and data quality to health workers themselves. Our co-creative, HCD approach focused the team’s work at the very point in the system where healthcare happens *before* it becomes data *about* that care’.^29^ The role of the national principal investigators in each country was instrumental in providing clinical credibility to the task before health workers.

## Conclusions

Our report illustrates a complex and resource-intensive process required to design a systemic intervention.

1. RHIS have to be designed by qualified designers who work under the paradigm that any attempt to improve RHIS should facilitate decision-making of frontline health workers in their daily, routine practice, be it clinical, public health or managerial decisions.
2. Everything is not possible and not equally important: it is not possible to ask health workers to invest their time in producing the best quality of care and also in producing and transmitting numerous data, unless necessary resources are made available.
3. A meaningful HCD has several components: a commitment to address real problems affecting a certain group of people; systematic and flexible process management; encompassing all research phases, from inception to interpretation; dealing with the uncertainty inherent to any creative process; enough resources, including time; and trust between team members with different skills.
4. Based on our conviction that HCD is not an optional ‘nice to have’ capacity in systems research, but rather a crucial component of the research, we call for researchers, designers, healthcare providers, healthcare authorities and funding agencies to propose and pilot quality standards for the implementation and reporting of HCD in global health. HCD may become a fundamental ingredient within the context of global health initiatives. HCD serves as one of the core components that underpins the development, implementation and evaluation of healthcare interventions.
5. Design organisations working with HCD in global health need to be very adaptive, active listeners to the needs of researchers, co-creation groups and health workers and have extensive experience in global health. HCD can bring most value when it facilitates decision-making across different disciplines.

## Supplementary material

10.1136/bmjgh-2025-019180 online supplemental file 1

10.1136/bmjgh-2025-019180 online supplemental file 2

10.1136/bmjgh-2025-019180 online supplemental file 3

## Data Availability

All data relevant to the study are included in the article or uploaded as supplementary information.
