# Supplementary material for: How to design decision-support tools for primary healthcare using a human-centred design approach: the processes and experience of PHISICC in three Sub-Saharan countries
Source: BMJ Glob Health. 2026 Jan 14;11(1):e019180. doi: 10.1136/bmjgh-2025-019180 (PMC12815237; doi:10.1136/bmjgh-2025-019180)
Supplement: online supplemental file 1 [file bmjgh-11-1-s001.pdf]

SUPPLEMENT 1: Field work illustrations and outputs: illustrations of challenges encountered, solutions contemplated, solutions applied and feedback received about prototype PHISICC tools

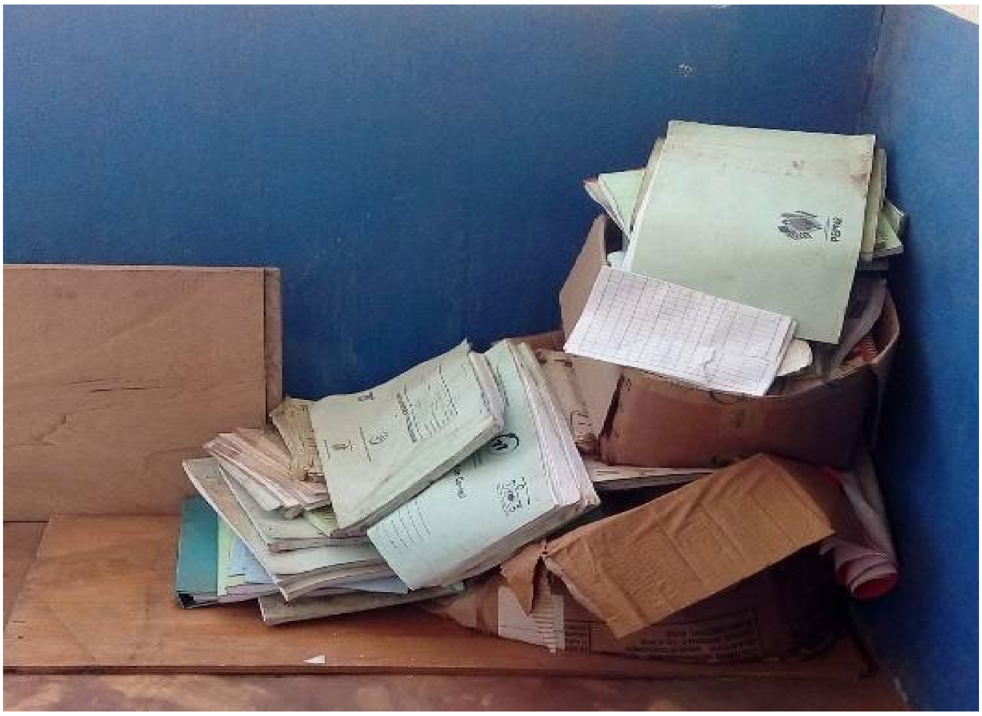

Figure 1. Office space with health information tools.

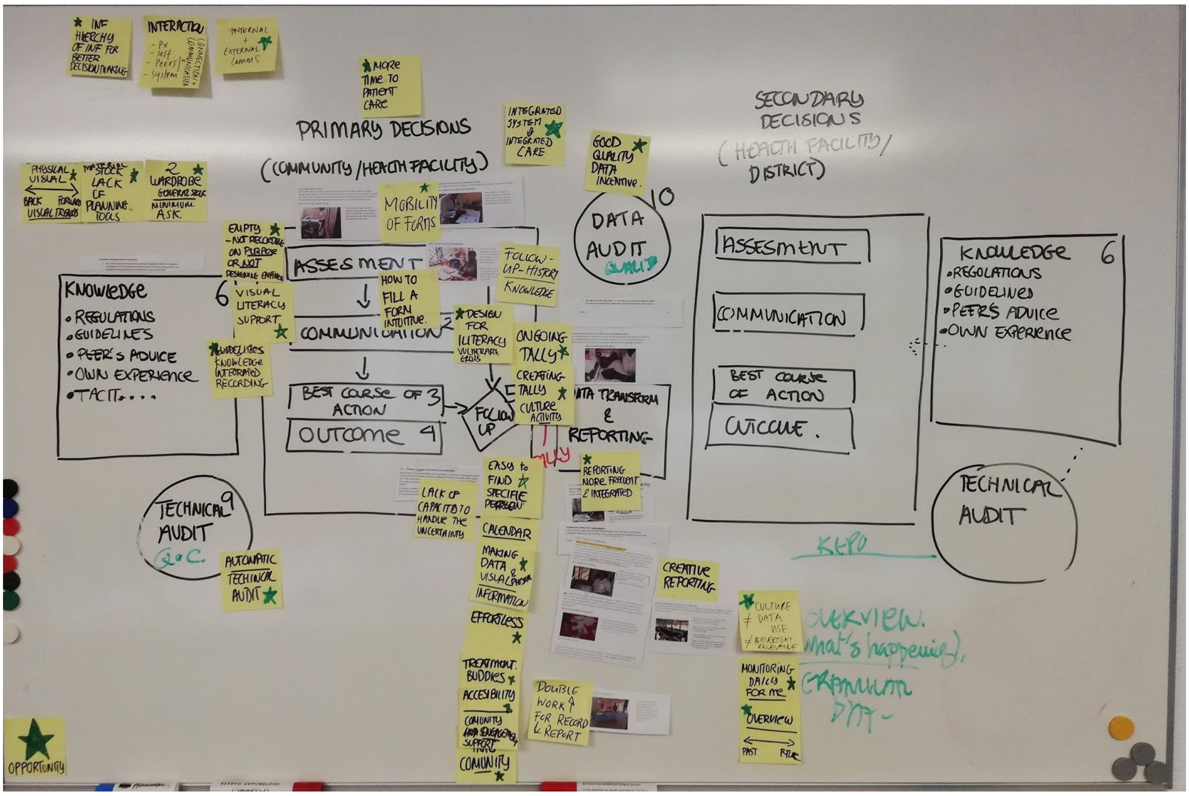

Figure 2. Key findings mapped into the Research Synthesis Framework.

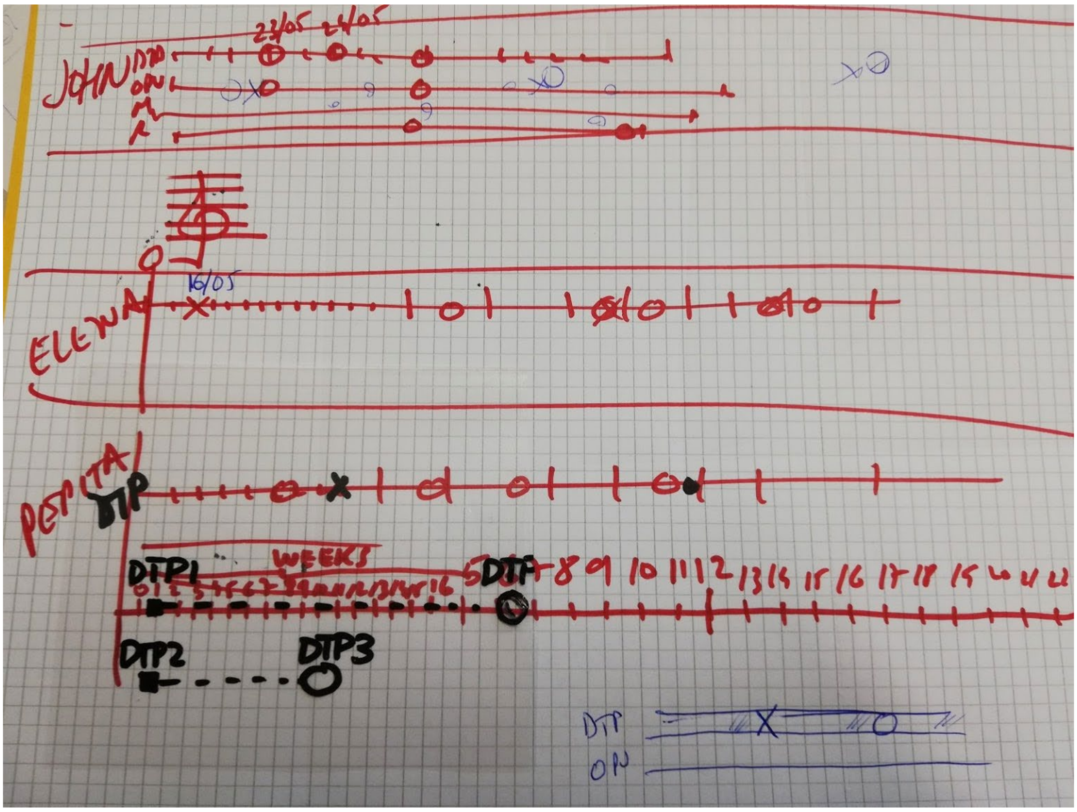

Figure 3. First sketches of the vaccination registry.

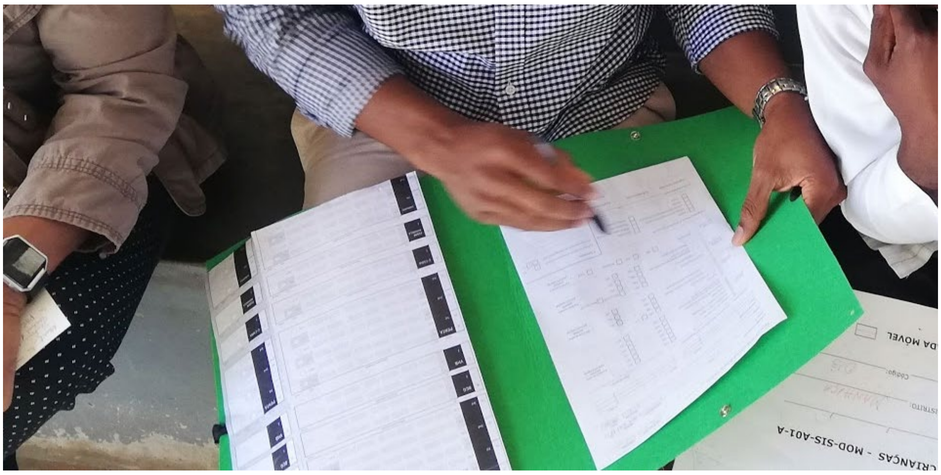

Figure 4. Nigeria co-creation group conducting user-testing in a health facility in Mozambique, where they travelled.

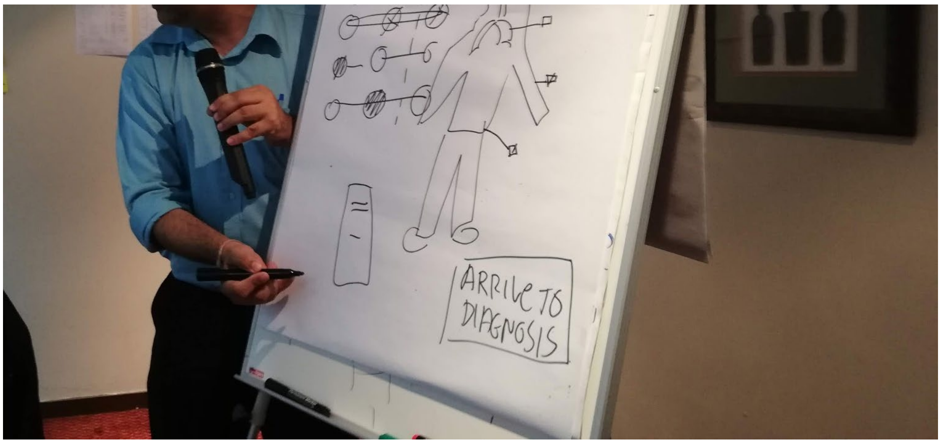

Figure 5. Co-creation session where the team discussed the need for more simple visual clues for decision-making for Sick Child registry.

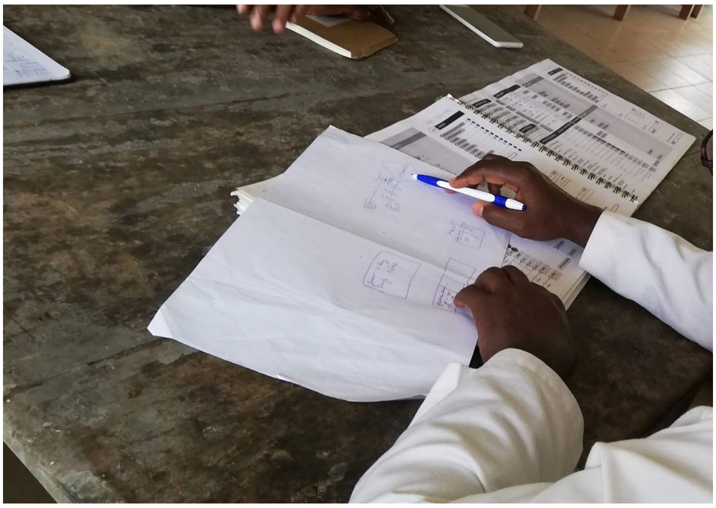

Figure 6. Frontline worker suggesting improvements for the Sick Child registry.

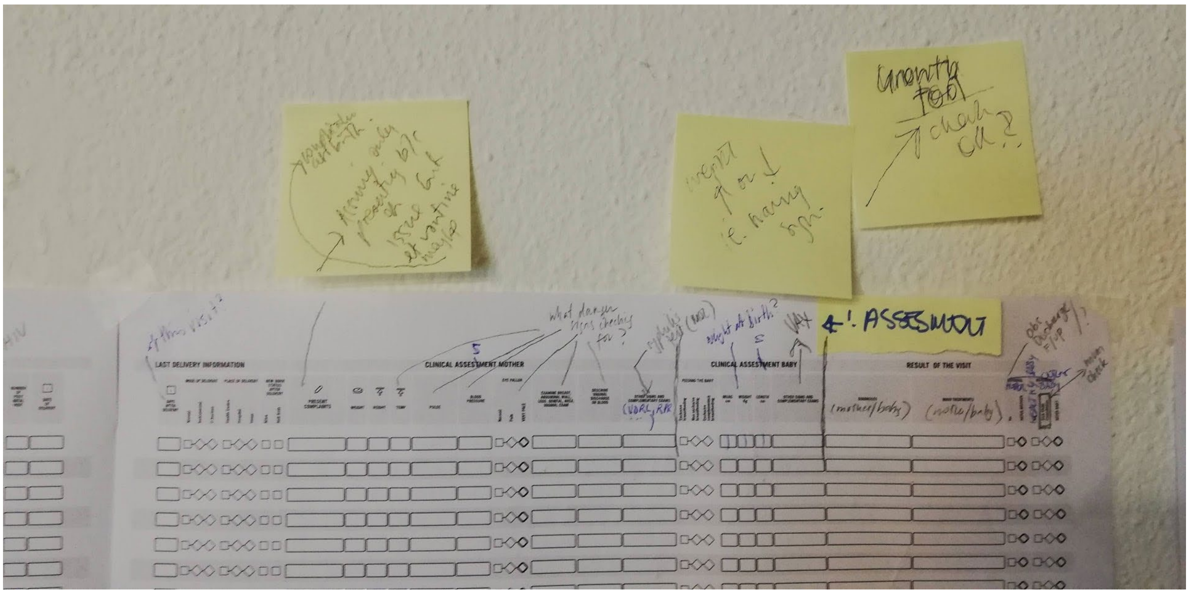

Figure 7. Redesign process for the post-natal care registry.
