# Supplementary material for: How to design decision-support tools for primary healthcare using a human-centred design approach: the processes and experience of PHISICC in three Sub-Saharan countries
Source: BMJ Glob Health. 2026 Jan 14;11(1):e019180. doi: 10.1136/bmjgh-2025-019180 (PMC12815237; doi:10.1136/bmjgh-2025-019180)
Supplement: online supplemental file 2 [file bmjgh-11-1-s002.pdf]

1 CHILD

IDENTIFY THE CHILD

SURNAME, FIRST NAME

ID NUMBER

ADDRESS

SEX
- [ ] Male
- [ ] Female

IDENTIFY THE CAREGIVER

SURNAME, FIRST NAME

RELATIONSHIP

PHONE NUMBER

DATE OF BIRTH
D D M M Y Y

	AT BIRTH	M1	.	.	.	M2	.	.	.	M3	.	.	.	M4	.	.	.	M5	M6	M7	M8	M9	M10	M11	M12	M13	M14	M15	M16	M17	M18	M19	M20	M21	M22	M23	M24
		W1	W2	W3	W4	W5	W6	W7	W8	W9	W10	W11	W12	W13	W14	W15	W16	.	.	.	.	.	.	.	.	.	.	.	.	.	.	.	.	.	.	.	.
BCG																																					
HBV																																					
POLIO																																					
DTP-CONTAINING VACCINE																																					
PCV																																					
ROTA																																					
MEASLES																																					
MEN A																																					
YELLOW FEVER																																					
VITAMIN A																																					

OUTCOME AT 12 MONTHS
- ◇ Partially vaccinated
- [ ] Fully vaccinated

2 CHILD

IDENTIFY THE CHILD

SURNAME, FIRST NAME

ID NUMBER

ADDRESS

SEX
- [ ] Male
- [ ] Female

IDENTIFY THE CAREGIVER

SURNAME, FIRST NAME

RELATIONSHIP

PHONE NUMBER

DATE OF BIRTH
D D M M Y Y

	AT BIRTH	M1	.	.	.	M2	.	.	.	M3	.	.	.	M4	.	.	.	M5	M6	M7	M8	M9	M10	M11	M12	M13	M14	M15	M16	M17	M18	M19	M20	M21	M22	M23	M24
		W1	W2	W3	W4	W5	W6	W7	W8	W9	W10	W11	W12	W13	W14	W15	W16	.	.	.	.	.	.	.	.	.	.	.	.	.	.	.	.	.	.	.	.
BCG																																					
HBV																																					
POLIO																																					
DTP-CONTAINING VACCINE																																					
PCV																																					
ROTA																																					
MEASLES																																					
MEN A																																					
YELLOW FEVER																																					
VITAMIN A																																					

OUTCOME AT 12 MONTHS
- ◇ Partially vaccinated
- [ ] Fully vaccinated

3 CHILD

IDENTIFY THE CHILD

SURNAME, FIRST NAME

ID NUMBER

ADDRESS

SEX
- [ ] Male
- [ ] Female

IDENTIFY THE CAREGIVER

SURNAME, FIRST NAME

RELATIONSHIP

PHONE NUMBER

DATE OF BIRTH
D D M M Y Y

	AT BIRTH	M1	.	.	.	M2	.	.	.	M3	.	.	.	M4	.	.	.	M5	M6	M7	M8	M9	M10	M11	M12	M13	M14	M15	M16	M17	M18	M19	M20	M21	M22	M23	M24
		W1	W2	W3	W4	W5	W6	W7	W8	W9	W10	W11	W12	W13	W14	W15	W16	.	.	.	.	.	.	.	.	.	.	.	.	.	.	.	.	.	.	.	.
BCG																																					
HBV																																					
POLIO																																					
DTP-CONTAINING VACCINE																																					
PCV																																					
ROTA																																					
MEASLES																																					
MEN A																																					
YELLOW FEVER																																					
VITAMIN A																																					

OUTCOME AT 12 MONTHS
- ◇ Partially vaccinated
- [ ] Fully vaccinated

PHISICC - minibeta - Nigeria - 2019
NOT FOR DISTRIBUTION

1 IDENTIFY THE CHILD

TODAY´S DATE: D D M M Y Y

IDENTIFY THE CHILD

SURNAME, FIRST NAME | ID NUMBER

DATE OF BIRTH: D D M M Y Y | AGE: YEARS MONTHS

SEX: ☐ Male ☐ Female

ADDRESS

IDENTIFY THE CAREGIVER

SURNAME, FIRST NAME | RELATIONSHIP

PHONE NUMBER

PAST CLINICAL HISTORY

ANNOTATE ANY OTHER MEDICAL, SURGICAL OR OTHER ANTECEDENTS

PRESENT CONDITION

PRESENTING COMPLAINTS (COMPLAINT AND DURATION)

IS THE CHILD A TB PATIENT CONTACT?	No	Yes

REGISTER LOCATION - TB CASE: BOOK | PAGE

CHILD´S HIV STATUS (ONLY IF MOTHER HIV POSITIVE OR UNKNOWN)

HIV	Negative	Unknown	Exposed	Confirmed

REGISTER LOCATION - HIV: BOOK | PAGE

ARV TREATMENT STARTED	Not needed	Treatment delayed	Treatment started

2 KEY MEASUREMENTS

MEASURE

TEMPERATURE	°C
BREATH RATE	BREATHS / MIN
HEART RATE (PULSE)	BEATS / MIN
WEIGHT	Kg
LENGTH / HEIGHT	cm

3 CHECK CRITICAL DANGER SIGNS

IS THE CHILD 0 TO 2 MONTHS OLD? ☐ No (skip) ☐ Yes (complete section)

WHAT IS THE BABY'S TEMPERATURE?	Normal	LOWER THAN 35.5 °C	HIGHER THAN 37.5°C
CONSIDER	-	REFERRAL	REFERRAL

COMPLETE FOR ALL CHILDREN

IS THE CHILD SLEEPY, LETHARGIC OR UNCONSCIOUS?	No	YES
DOES THE CHILD HAVE CONVULSIONS?	No	YES
IS THE CHILD UNABLE TO DRINK OR BREASTFEED?	No	YES
DOES THE CHILD VOMIT EVERYTHING?	No	YES
CONSIDER		VERY SEVERE DISEASE

CHECK RESPIRATORY SIGNS IN ALL CHILDREN

CAN YOU HEAR A SOUND WHILE BREATHING (WHEEZING OR OBSTRUCTION)?	No	Moderate	SEVERE
ANY CHEST INDRAWING?	None	Yes	YES IN BABY 2 MONTHS OR LESS
BREATH RATE (CHECK MEASUREMENTS)	Normal	Fast *	FAST IN BABY 2 MONTHS OR LESS *
CONSIDER DIAGNOSIS	COUGH OR COLD	PNEUMONIA	SEVERE PNEUMONIA

*BREATH RATE ABNORMAL VALUES

0 to 2 months of age: more than 60 breaths / minute
2 to 12 months of age: more than 50 breaths / minute
12 months up to 5 years of age: 40 breaths / minute

4 SPECIFIC CONDITIONS

ANY LOOSE STOOLS OR DEHYDRATION? ☐ No (skip) ☐ Yes (complete section)

WHAT IS THE LEVEL OF CONSCIOUSNESS?	Normal	Slightly sleepy	VERY SLEEPY (LETHARGIC)
IS THE CHILD ABLE TO DRINK?	Normally	Hardly	NOT AT ALL
HAS THE CHILD SUNKEN EYES?	No	Slightly	YES
SKIN PINCH DURATION (SECONDS)	Quick	1 to 2 Seconds	2+ SECONDS
CONSIDER DIAGNOSIS	MILD DEHYDRATION	MODERATE DEHYDRATION<br>2 of any above	SEVERE DEHYDRATION<br>More than 2 above

ANY FEVER? ☐ No (skip) ☐ Yes (complete section)

IS THERE ANY DANGER SIGN PRESENT IN STEP 3?	No		YES
HAS THE CHILD STIFF NECK?	No		YES
MALARIA TEST	Negative	Positive or not done	
CONSIDER DIAGNOSIS	FEBRILE DISEASE	MALARIA	VERY SEVERE FEBRILE DISEASE

IF MEASLES NOW OR RECENTLY AND EYE PROBLEM?	No	Pus Draining	CLOUDING OF CORNEA
IF MEASLES NOW OR RECENTLY AND MOUTH ULCERS?	No	Mild	DEEP OR EXTENSIVE
CONSIDER DIAGNOSIS	MEASLES	COMPLICATED MEASLES	SEVERE COMPLICATED MEASLES

ANY PALLOR OR BLOOD ISSUE? ☐ No (skip) ☐ Yes (complete section)

HAS THE CHILD PALMAR (OR EYE) PALLOR?	No	Some	SEVERE
CONSIDER DIAGNOSIS	NO ANEMIA	MODERATE ANEMIA	SEVERE ANEMIA

ANY SIGNS OF MALNUTRITION? ☐ No (skip) ☐ Yes (complete section)

HAS THE CHILD SWOLLEN FEET (OEDEMA)?	No			YES
WEIGHT FOR LENGTH / MUAC (CLINICAL NOTE)	More than -2z<br>12.5 cm or more<br>Green	-2z to -3z<br>11.5 to 12.5 cm<br>Yellow		LESS THAN -3Z<br>11.5 CM OR LESS<br>RED
UNABLE TO DRINK?			No	YES
CONSIDER DIAGNOSIS	NO MALNUTRITION	MODERATE MALNUTRITION	UNCOMPLICATED SERVERE MALNUTRITION	COMPLICATED SERVERE MALNUTRITION

ANY JAUNDICE (YELLOWISH SKIN)? ☐ No (skip) ☐ Yes (complete section)

PALMS AND SOLES YELLOW	No		YES
YELLOWISH SKIN ELSEWHERE	No	Only after 24 hours of life	WITHIN 24 HOURS OF LIFE
CONSIDER DIAGNOSIS	NO JAUNDICE	JAUNDICE	SEVERE JAUNDICE

OTHER SIGNS (CONSIDER FEEDING PROBLEMS IN LESS THAN 2 MONTHS OF AGE)

5 DECISIONS

DIAGNOSES - REVIEW YOUR FINDINGS ABOVE AND STATE THE DIAGNOSES

Consider TB, HIV, neglected diseases...

TREATMENTS - CONSIDER PRE-REFERRAL TREATMENTS (PAGE 3)

NEED TO UPDATE VACCINATIONS?	Up to date	Out of date

OUTCOME	Treatment	TB visit	HIV visit	Follow up	REFERRAL

NEXT APPOINTMENT IN: DAYS

TALLY DONE ☐ | HEALTH WORKER SIGNATURE

PHSICC - minibeta - Nigeria - 2019
NOT FOR DISTRIBUTION
