# Supplementary material for: How to design decision-support tools for primary healthcare using a human-centred design approach: the processes and experience of PHISICC in three Sub-Saharan countries
Source: BMJ Glob Health. 2026 Jan 14;11(1):e019180. doi: 10.1136/bmjgh-2025-019180 (PMC12815237; doi:10.1136/bmjgh-2025-019180)

EVOLUTION OF FORMS’ DESIGN

Three partial snapshots of the *sick child form* focusing on respiratory signs, to exemplify the evolution of versions across the design process.

(1) Early version

Symptoms and signs were not linked to diagnoses yet.

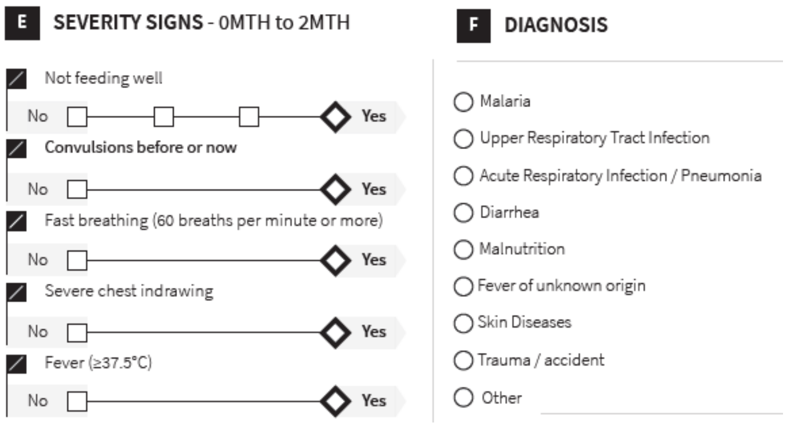

(2) Intermediate version

Symptoms and signs are linked to diagnoses, but the spatial distribution is not solved.

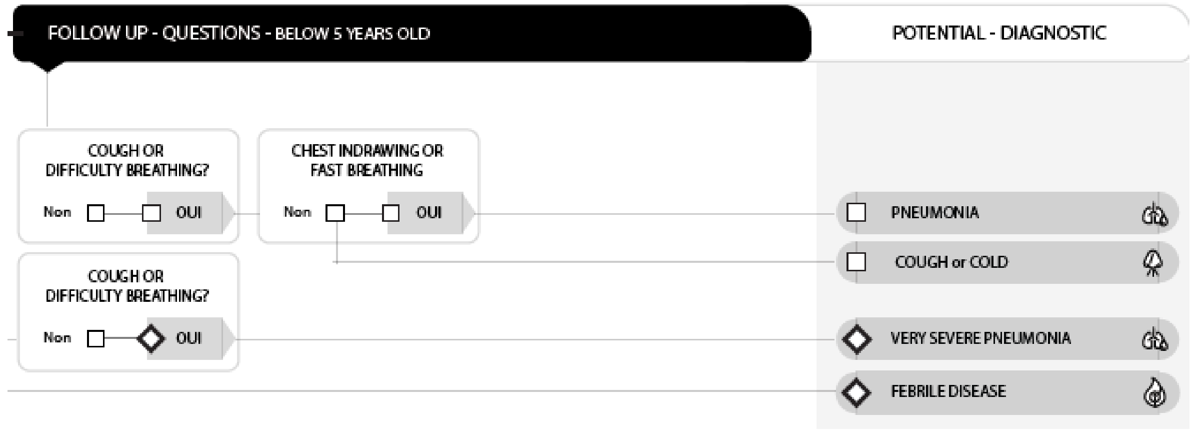

(3) Definitive version

Symptoms and signs are linked to the diagnoses and spatially arranged with severity classification.

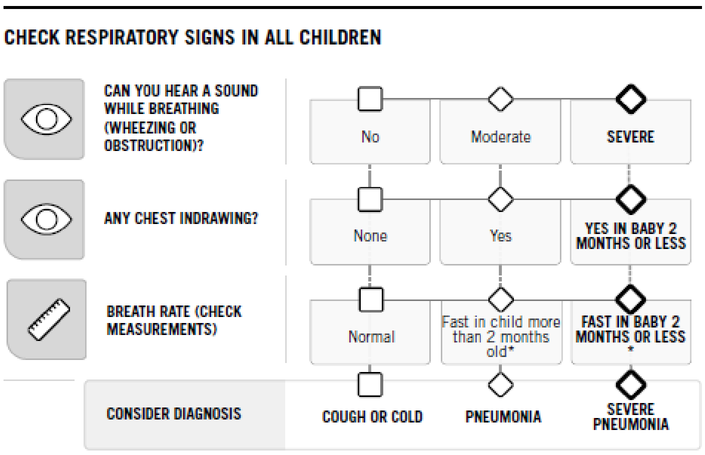

Supplement: online supplemental file 3 [file bmjgh-11-1-s003.pdf]
